# Poly-truth, or the limits of pluralism: Popular debates on conspiracy theories in a post-truth era

**DOI:** 10.1177/09636625221092145

**Published:** 2022-04-28

**Authors:** Jaron Harambam, Kamile Grusauskaite, Lars de Wildt

**Affiliations:** VU University Amsterdam, The Netherlands; KU Leuven, Belgium

**Keywords:** conspiracy theories, disinformation, epistemology, expertise, experts, pluralism, poly-truth, post-truth, public debate

## Abstract

Conspiracy theories are central to “post-truth” discussions. Official knowledge, backed by science, politics, and media, is distrusted by various people resorting to alternative (conspiratorial) explanations. While elite commentators lament the rise of such “untruths,” we know little of people’s everyday opinions on this topic, despite their societal ramifications. We therefore performed a qualitative content analysis of 522 comments under a Dutch newspaper article on conspiracy theories to study how ordinary people discuss post-truth matters. We found four main points of controversy: “habitus of distrust”; “who to involve in public debates”; “which ways of knowing to allow”; and “what is at stake?” The diverging opinions outline the limits of pluralism in a post-truth era, revealing tensions between technocratic and democratic ideals in society. We show that popular opinions on conspiracy theories embody more complexity and nuance than elite conceptions of post-truth allow for: they lay bare the multiple sociological dimensions of *poly-truth*.

## 1. Introduction

Conspiracy theories are central to contemporary public discourse. No longer in the dark extremist margins of society, these allegations, usually of some secret cabal operating behind the everyday screen of reality, now take center stage in various societal debates. Think of former US president Donald Trump, or his following propelled by Q-Anon theories to fight against a nefarious “deep state,” or the so-called *anti-vaxxers* to *9/11-truthers* and climate skeptics, who all challenge the “official” accounts of reality. This widespread distrust toward mainstream epistemic authorities and the knowledge they produce is a serious concern for many who fear the dissolution of a shared truth, spiraling polarization, and the demise of democratic societies (d’Ancona, 2017; Rosenblum and Muirhead, 2020; Sunstein, 2018). The coronavirus pandemic only aggravated and intensified this socio-cultural development often dubbed post-truth: much of our understanding of the virus, how it impacts our bodies and societies, and what to do about it, is contested from various pockets in society (Harambam, 2020b). It is not hard to imagine that the pervasiveness of these alternative accounts of officially sanctioned truths strongly influences people’s opinions on public institutions such as the media, politics, and science and on the functioning of democratic societies. They also lead to heated personal and public debates, and societal polarization.

Yet we find very little academic research on how people regard the presence of conspiracy theories in the public sphere. Over the last two decades, a burgeoning research field on conspiracy theories emerged (Butter and Knight, 2020; Douglas et al., 2019; Drążkiewicz and Harambam, 2021; Uscinski, 2018). Scholars from many different academic disciplines study conspiracy theories: What are they? Where do they come from? Which people adhere to them? How many people believe them? What meaning do they have? How do they function? What consequences do they have? And so on. Such research centers, rather logically, around conspiracy theories/-ists. However, this exclusive focus obscures the fact that conspiracy theories operate in societal context, affecting many more people beyond conspiracy theorists alone. Elite opinions of commentators in media, politics and science on the dangers of conspiracy theories abound, but what about people’s everyday appreciations of these competing truth claims? They are remarkably invisible in academic research.

In this article, we therefore study public opinions of conspiracy theories to empirically show how people think and talk about them, and ultimately, to grasp the multiple cultural meanings of these “stigmatized forms of knowledge” in contemporary Western societies (Barkun, 2006; Birchall, 2006). To pursue this research question, we conducted a qualitative content analysis of 522 comments by Dutch speakers below a newspaper article about conspiracy theories published in May 2020 on the largest online news platform in the Netherlands. The news article consists of an interview with the main author of this article and discusses the contemporary popularity of conspiracy theories, and whether and how we should engage with conspiracy theorists. As a mainstream popular news outlet, *NU.nl* draws a diverse crowd, making the sample fit to obtain a general overview of the variety of public opinions on conspiracy theories. More precisely, it gives insight into the various positions and logics of the public debate on conspiracy theories in the midst of the SARS-CoV-2 pandemic when various alternative perspectives on the crisis were rampant. Quantitative survey research makes visible the amount of people endorsing various conspiracy theories (Uscinski, 2018), but gauging people’s opinions of conspiracy theories is perhaps even more important to understand their cultural and societal significance.

Both in private and public contexts, conspiracy theories appear a divisive topic. Many point to the personal and public harms conspiracy theories may cause: from interpersonal and intergroup conflicts and societal polarization, to increased prejudice toward other groups, harmful health choices, science denial, lower political engagement, extremism, radicalization, and sometimes even violence (Byford, 2011; Cassam, 2019; Douglas et al., 2019). Such commenters argue that conspiracy theories pose clear dangers to various public institutions as their legitimacy and authority are called into question (Kakutani, 2018; Lewandowsky et al., 2017; Sunstein, 2018). But conspiracy theories also connect people, give meaning to experienced disparities and corruption in society, and may force mainstream institutions and authorities to be more accountable (Dentith, 2018; Fenster, 2008; Harambam and Aupers, 2017). Conspiracy theories are simply not as straightforward as is often portrayed. Indeed, we might ask: if we listen to what everyday people say about conspiracy theories, what do we hear and learn?

## 2. Conspiracy theory as the epitome of post-truth

Scholarly analyses of conspiracy theories can be divided into two opposing camps. On one hand, there are those that pathologize conspiracy theories: building on seminal works of Karl Popper (2013) and Richard Hofstadter (2012), these scholars regard conspiracy theories to be flawed, irrational, and dangerous understandings of reality. They argue that conspiratorial ideas are reminiscent of outdated religious worldviews and embody pseudoscience; they are the delusional thought of disturbed, overly paranoid minds; and go against the (preferred) political virtues of moderation, deliberation, and consensus (Barkun, 2006; Byford, 2011; Cassam, 2019; Pipes, 1997; Sunstein, 2018). By contrast, other scholars, mostly from a more anthropological bent, take a less normative approach to better understand their cultural significance (Harambam, 2020a). They relate conspiracy theories to the complexities of today’s globalized, hyperreal and risk-saturated world, and see them as “necessary,” “creative,” “logical,” “appropriate,” “tactical,” and even “productive” responses to technological, social, and cultural change (Birchall, 2006; Dean, 1998; Fenster, 2008; Knight, 2000; Melley, 2000). Furthermore, they argue that what constitutes a conspiracy theory is a consequence of societal power asymmetries which enable their stigmatized labeling and societal exclusion (Bjerg and Presskorn-Thygesen, 2017; Bratich, 2008; Husting and Orr, 2007; Pelkmans and Machold, 2011). Prevalent academic distinctions between irrational conspiracy theories and rational science can therefore be seen as professional boundary work, upholding the epistemic authority of science (Harambam and Aupers, 2015).

The latter cultural perspective seems remarkably absent in most contemporary post-truth discussions. Conspiracy theories are commonly juxtaposed with science and factual knowledge (Biesecker, 2018), and put on par with equally slippery concepts such as fake news and mis/disinformation, which together come to designate what post-truth is about (Lewandowsky et al., 2017; McIntyre, 2018). In this new era, the argument goes, shared agreements on what constitutes facts and truths are ignored or dismantled, and a gratuitous usage of those terms in the public sphere is no longer taken to be problematic (Kakutani, 2018). Appeals to emotion and personal beliefs appear more influential in shaping public opinion and political debates than so-called objective facts or expert knowledge (Bennett and Livingston, 2018). Various actors make claims on truth without empirical support and discard inconvenient facts simply by calling them false (Rosenblum and Muirhead, 2020), and their followers continue to support such actors even when their claims are widely debunked (Lewandowsky et al., 2020). These developments seriously alarm academic scholars, media professionals, and politicians alike: they point to dangerous consequences of the demise of reason and facts in public debate (Kakutani, 2018; Wardle and Derakhshan, 2017). Such nefarious forms of untruthful information delegitimize science and public institutions, may support and motivate anti-democratic politics, and often fuel racist ideologies and actions (Berlet, 2009; Byford, 2011; Pollard, 2016).

The dominant diagnostic response to post-truth issues is the reinstalment of the modernist legitimation narrative of science (Harambam, 2021): conspiracy theories (and other forms of “inferior” information) should be debunked (fact-checked) and/or banned from the public sphere (content moderation), while people should listen to legacy media and scientific experts again (Drążkiewicz and Harambam, 2021; Wardle and Derakhshan, 2017). While some *Science and Technology Studies* scholars have rightfully pointed to the confluence of values, politics, and facts in scientific *and* public knowledge (Fuller, 2018; Jasanoff and Simmet, 2017; Marres, 2018), most post-truth discourse presents clear-cut distinctions between the esteemed objective realm of facts, science, and reason and the dangerous subjective realm of emotions, ideology, and irrationality.

The point here is not to argue which academic perspective is right, but to show how different academics draw different boundaries around conspiracy theories and ascribe different meanings to them. The interesting observation to make is that while diversity is much advocated by academics and elite commenters across different domains (be it about age, race/ethnicity, gender, religion, or culture), this does not seem to fly for people who have other ideas about the truth. Key to democratic “open societies” may be the plurality of opinions, ideologies, and media to foster a productive confrontation of ideas (Popper, 2013), but in contemporary post-truth debates this ideal is not easily found. In fact, clear limits on free speech are enforced when it comes to truth, while modern distinctions between facts and non-facts (values, feelings, opinions) are upheld. Does this resonate with a general public?

Here, we follow a cultural-sociological approach to study what boundaries everyday people draw when they speak about conspiracy theories in the public sphere. There has been remarkably little research on people’s appreciations of dubious information in the public sphere: most scholarly analyses of post-truth are either theoretical or essayistic (Kakutani, 2018; McIntyre, 2018), or focus on the media ecosystem (Bennett and Livingston, 2018). Some scholars did, however, study the public opinion and perceptions of fake news in the US context, finding that these were polarized along partisan identities (Tong et al., 2020), while others showed people’s reactions to fake news and their effects on interpersonal relationships (Duffy et al., 2020). Yet, a systematic and in-depth analysis of people’s everyday ideas and conversations about these post-truth matters is wanting. The comments we analyze in this article display people’s ordinary discussions and are informed by their everyday encounters with conspiracy theories/-ists. Do they believe that we should do something about their popularity or are they seen as a valuable contribution to pluralist societies? Now that elite appreciations of conspiracy theories/-ists and post-truth abound, and inform much public debate and institutionalized policy, we wonder how that resonates with the opinions of a more general and diverse public. Is the post-truth condition perhaps an elite concern?

## 3. Method, data, and analysis

We performed a qualitative content analysis on the comments section below the interview article with the main author on *NU.nl*. The interview was about the contemporary popularity of conspiracy theories, how their stigmatization may engender radicalization, and whether we should include their propagators in public debates. Such discussions are increasingly held in the Netherlands where conspiracy theories thrive, just as other North-Western European countries, in various pockets of society (from leftist urban hipsters to right-wing extremists) and across different demographic groups (Butter and Knight, 2020; Harambam, 2020a). Instead of trying to contain it, the Dutch media find it important to discuss those matters publicly, and various news reports, documentaries, and TV shows on the topic emerge. The comments were rich and detailed enough to warrant a sociological content analysis, while *NU.nl* is a suitable general news platform to gauge a wide variety of opinions. *NU.nl* is the largest online news platform in the Netherlands at 7 million users (>45% of the Dutch population), free of charge to readers, and posts original journalistic content including news, opinions, and interviews from various viewpoints. It furthermore has an active comments section below articles, which is rare in Dutch news media nowadays (Hille and Bakker, 2014). *NU.nl* has a moderation team, which removes comments that go against their guidelines, which mostly come down to hate speech. Nonetheless, NU.nl shows when comments are removed, and most were recovered by us, as shown in our publicly available dataset.^1^ Self-selection may obviously at play: commenters may be more invested in the subject matter or have more outspoken opinions. However, in qualitative analyses, representativeness (of the general population or *NU.nl* readership) is not the goal, but instead a wide diversity of standpoints is. Although readership demographics are not shared by *NU.nl*, Dutch online news readership in general is high and well-distributed across different demographic groups. The annual *Digital News Report Nederland 2021* indicates that *NU.nl* is seen by readers across age groups as more diverse than other news platforms (Newman, et al., 2021). The resulting dataset consists of all 522 comments until the discussion was closed three days later. Informed consent was not deemed necessary under *NU.nl’s* terms and conditions (De Wildt and Aupers, 2020).

Our qualitative analysis was done inductively, loosely following the principles of Grounded Theory (Charmaz, 2006). We focused on finding common themes and patterns in the data to incrementally develop higher level concepts that are (Weberian) “ideal-typical reconstructions” (Inglis and Almila, 2016: 33). We used qualitative data analysis software Atlas.ti to systematize our analysis throughout the four iterations from data to concepts. From the start, we worked closely to ensure analytical consistency: first, to arrive at a coding book, the three authors independently coded 20% of all comments. We then jointly discussed the meaning, process, and logic of our codes, resulting in 87 open codes (e.g. “conspiracy theories irrational,” “no conversation possible,” or “dogmatic scientists”), with which we then analyzed a third of the data each. Second, through axial coding, we subsumed and connected codes to condense them to a code tree of 34 concepts (e.g. “multiple voices important” or “distrusting elites”). A third and fourth interpretative round regrouped and subsumed those codes into 10 and later 4 theoretically informed “supercodes” which now form the backbone of the following results section.

## 4. Popular debates on conspiracy theories in a post-truth era

Our analysis showed a wide range of opinions about conspiracy theories, and their role in public debates. Discussion topics ranged from what conspiracy theories are (and how to draw lines between legitimate critiques of dominant truths and dogmatic accusations of conceit), how people experience conspiracy theorists, what forms of knowledge they find legitimate, which epistemic sources to follow and why, and what the societal consequences of these choices would be. We now present this variety along the four main categories of dispute: “habitus of distrust,” “actors,” “knowledge,” and “consequences.”

### Habitus of distrust: How to appreciate established truths?

On the most abstract level, commenters discussed the rationality of conspiracy theorists’ ‘habitus of distrust’: a deep-seated suspicion of elites, institutions, and official truths. While some argue that this attitude to the world is too extreme and poisonous, others view this as legitimate, logical, and even useful.

The first group of commenters views distrust in elites and public authorities as irrational and criticizes conspiracy theorists for their paranoid style of thinking. They question the need to challenge existing truths: if something has been considered true, stood the test of time, and “had the same outcome before” (Kbg), why contest it? Instead, they conservatively trust established knowledge: “the problem with conspiracy theorists is that many of the things they contest have already been studied and proved otherwise” (William). One commenter draws an analogy with parenting and compares trust in mainstream institutions to trusting one’s parents:

Did your mother cite sources? Did you ever double-check her findings growing up? Or instead, did you trust that she is speaking the truth because she is your mother and had your best interests at heart? Being critical is allowed, but having a little faith in others isn’t bad either. (Umuriel)

Conspiracy theorists thus go too far in their distrust of institutions that actually merit more credit. But they also go too far in their creativity to imagine ever greater schemes of deception. Such commenters point to underlying parsimony principles as reasons to trust established knowledge: “an important measure is Ockham’s razor, freely translated: you should need as little as possible to explain an observation” (hs5), and established truths simply have “fewer assumptions to explain the world. That doesn’t mean that conspiracies don’t exist, but that they would be improbable” (JHP). They hold that “there may be an infinite amount of explanations possible, but we need to stick to the most plausible ones for which there is evidence” (Klopger). According to these commenters, conspiracy theorists’ questioning of established knowledge arises from a habitus of distrust that is both overly suspicious and illogical.

Other commenters highlight the opposing position that a habitus of distrust is a logical attitude to understand today’s world. First, they argue that it would be foolish to assume that expert knowledge is the “truth” merely because it has been accepted as such. A common example to illustrate this argument is the historical debate over the spherical earth: “there was once a conspiracy theory that the earth would be round instead of flat. You could be hanged for believing it, but it led to valuable insights” (Marc_Heijdra). Pointing to the dynamics of contemporary expert knowledge production, they furthermore contend that once a “truth” emerges, other experts build on it “without replicating the underlying research,” which is problematic because “a certain vision is increasingly taken as true, simply because it is confirmed by others” (Saksaf). Commenters argue that those producing established truths may have interests in preserving the status quo: “where there is power, people can manipulate reality and render other truth-seekers unbelievable” (Cor_Arnhem), “politicians hide the truth and make it difficult to find” (Maarten1), “many scientists prefer their ego to science” (Viggo), and “scientists are so full of their truths that they refuse to study divergent ideas or dismiss them because that would prove their truths wrong, which costs money! Money is what most of the scientific status quo is about” (Koen_de_Vos). These commenters are “often surprised about the lengths that people go to hold onto the official narrative” (Ray_Man). Instead, they argue that conspiracy thinking and a habitus of distrust is how new ideas are formed: “we should not by default assume that what the mass media tells us is true, and instead look for new (possible) answers” (Patricia_C).

Such commenters often base the rationality of distrust on the fact that “conspiracy is inherent to life” (PeterR_2), and that conspiracies have happened before, thus “how insane are conspiracy theories then?” (kos_kosso). They point to various examples of covert US state projects such as “Operation Northwoods, Project Sunshine, MK-Ultra or Tuskegee Syphilis Study” (display_name) or “the ‘legalized’ invasion of Iraq by Bush who had ‘proofs’ of weapons of mass destruction, which turned out to be a massive lie. Conspiracy theorists distrusted this narrative back then, but were discarded as ‘loonies,’ but who’s the fool now?” (Hokkie_Stick). But commenters mention more mundane yet widespread “price fixing, tax fraud and insider trading” (Cooknbacker), “how governments concealed the dangers of asbestos” (Keesdie), or industry fueled myths such as “drinking milk is healthy. Or tobacco. 30 years later things look very different. Distrust towards truths is just not misplaced” (Jacques_de_vogel). These people often illustrate their habitus of distrust as resulting from personal experiences: “conspiracy theorists are streetwise and often experienced injustice themselves, which made them realize that people are not always good” (Skunk83), and “one thing I learned in my career with the military and the UN: take rumors very seriously, since there is always some truth to them, even if it seems impossible. I always have that approach in discussions with conspiracy thinkers” (loko_loki). Another commenter agrees, conspiracy theories may seem extreme at first, but “they simply indicate alternatives to accepted points of view. And because such counter-narratives aren’t given space, they need to be magnified to make a statement” (Oneshe). Such commenters all agree that a habitus of distrust may simply be reasonable in a corrupt world in which conspiracies do actually happen.

### Actors: Who should have a say in public debate?

Many discussants argued that experts (academics, politicians, journalists) should be the ones to listen to since they have specialized knowledge and acknowledge their own limitations, while conspiracy theorists are irrational and uninformed. By contrast, others argued that a broader public should be included to widen the narrow perspective of experts: laypersons can be more creative and do not have ulterior motives as experts are suspected of having.

Those favoring experts in the public debate see conspiracy theorists as irrational people, basing their ideas not on “facts and science,” but “believ[ing] in higher powers” and “hav[ing] a weird form of logic” (Bigmama_here). They “are unwilling to listen and discuss, everything is already decided and is brought back to the military-industrial complex, Bilderberg group, Illuminati and/or Rothschild family” (De_Observant). Harry_Gl adds, they “do not ask questions (anymore), but are locked in their own answers,” and if they do ask questions, these “are opinions disguised as cynical questions” (PdC). There is after all “a big difference between conspiracy theorists and people with diverging opinions” (De_Observant). Conspiracy theorists, these people contend, suffer from “self-made delusions” (Kaaselot), “dreams and phantasms” (Umuriel), and “paranoid explanations” (hs5). One commenter even portrayed conspiracy theorists as mental patients that belong in psychiatric institutions rather than in public debate:

the more exotic their convictions, the clearer they are disturbed people that can be ignored. We’ve got great education and journalism, so no need to succumb to weird conspiracy theories. Whoever goes cuckoo needs personal attention from a psychiatrist. (PdC)

Moreover, these commenters hold, conspiracy theorists “show herd behavior by sharing populist statements” (Dit_vind_ook_ik_ervan), “traffic in ridiculous generalizations and superlatives” (Asimo), and “always blame the same people, lumping whole industries (pharmaceuticals) or companies (Monsanto and Shell) together as devilish” (Bart_de_Koning). They point to “the Dunning-Kruger effect” (Thijzer, Ria_Heijnsbroek, Rene_Fijnhart), referring to the cognitive bias through which incompetent people overestimate their own abilities (Kruger and Dunning, 1999), “which is the reason that exactly those with least knowledge of things are most convinced of being right” (Rene_Fijnhart).

Experts are different, “like the prime minister, the RIVM and OMT [Dutch Public Health Institutes] who acknowledged from the beginning that they do not know everything” (ligfries), or who “from the start (and still now) are open and transparent about the fact that much is uncertain and needs more research” (Umuriel). These commenters express the view that in our complex societies we should listen to experts because they are the most qualified people:

I am not scientifically educated. I won’t be able to read hundreds of scientific publications. But then my colleagues with the same background suddenly pretend they can, and suddenly pretend to find the truth? I can’t take that seriously. (Polygoon)

Another adds that they “*want* to understand the unknown, but I can only do so if an expert/scientist explains it to me. I trust scientists, I trust experts. Why? They studied and researched for years” (VanDijk). They disagree about which experts should have a say, just those who speak “from their own perspective, like a virologist looking only at the physical impact of viruses and not at their societal consequences” (Diederik_de_Vries), or beyond, since “that fosters thinking outside of the box” (Richard Duiventil). But in principle, such commenters conclude, experts should dominate public debates because only they have relevant expertise and experience.

However, many others argued for a wider inclusion of laypeople and their opinions in public debates, including those that may seem outlandish and conspiratorial. Such commenters argue that experts are biased and may have ulterior motives, while the general public is independent. Such commenters wrote that:

focusing blindly on what science says may lead one to miss much and puts us on a dangerous track. We need both, a golden mean. It’s a mistake to uncritically follow scientists. Science is beautiful, it brings us much. But it is dangerous when people say: “if we can’t prove it, it does not exist.” (Thomas_Van_der_Langen)

Experts should therefore be more humble, and “doubt their own truths. I am afraid science passed that station long ago. Contemporary science is full of its own truths and discards other opinions directly” (Koen_de_Vos). Some urge experts therefore to be more courageous, saying that “good science dares to look beyond its boundaries. Much is achieved that way” (Caroline_Danon). Commenters point to potential ulterior motives, writing that “scientists have the tendency to adjust their research to the wishes of funders” (Viggo), and “often have interests in certain results” (Saksaf). Commenters often emphasize governmental corona-mitigation measures, which are seen as insufficiently substantiated or unconstitutional: “no one knows how and why measures are taken, which feeds speculators and conspiracy theorists” (Frances1). Especially the responsible public health institutes are distrusted, saying “there is zero transparency and new research is not (openly) incorporated. So yeah, then you get the feeling that there is a stubborn group of scientists dictating and not listening. That’s how people lose their trust” (DeWaarheidabc).

In contrast to experts, laypeople are attributed with having a broader view, giving their voice significance even though they may not align with mainstream (expert) narratives. This is beneficial because “that way you see more” (Pieter_de_Vries01010), “listening to people with ‘strange’ ideas enriches your own world and makes you see things differently” (Zwolse_Zelfstandige_Penvoerder), and “making space for alternative perspectives can be very productive, especially in a crisis where nobody holds the truth” (Matthijs_Grit). After all, “those with the least knowledge may actually ask the best questions” (robielienios), and “it is important to find out why people think in a certain way: that information can be very valuable” (Keesdie). Such commenters warn against a premature foreclosing of alternative views, arguing that “as soon as someone has diverging opinions, they get labelled conspiracy theorists and are dismissed as crazy, while their questions and insights are not that crazy at all” (kbg), and “it is extremely harmful to ridicule conspiracy theorists. Not all of them believe in Flat Earth or Corona by 5G” (Oke_dan). Such commenters warn against guilt-by-association, “the problem I often see is a focus on the craziest stories, which makes the rest look just as insane” (display_name).

Others problematize prevalent clear-cut distinctions between experts and conspiracy theorists, arguing that “it is a wrong assumption that conspiracy theorists would be people without substance. Many specialists, doctors, professors and other professionals are also dismissed as conspiracy theorists if their theory does not correspond with the generally accepted theory” (Gerard_van_der_Molen_efdacc). Given the fact that some conspiracy theorists advance rather farfetched ideas, commenters think we should

differentiate between those who ignore hard facts and let their own imagination go wild and those who use facts but come to a different conclusion. They are fine to discuss with, but are often overshadowed by the “conspiracy masses.” (Kaaselot)

Such commenters argue that “dogmatics are everywhere. There are those who will always cling to what the authorities say, while there are conspiracy theorists as well that are critical and who update their stance with new information” (Ynze_Bakker). So where to draw the line? Others explicitly value the imagination of conspiracy theorists, perhaps regardless of their truth value, saying “many are indeed a bit goofy and extreme in their craziness, but still. It happens often enough that you get a different perspective on matters, which make you rethink. The world would be a boring place without conspiracy theorists” (loko_loki).

### Knowledge: Which ways of knowing should we allow?

Whether by experts or conspiracy theorists, which truths should be allowed as part of the public debate? For some commenters, expert knowledge is legitimate because it is produced according to strict methodological procedures, and is based on factual evidence, so who are laymen to contest it? Others emphasize the value of intuition and experience as valid ways of knowing beyond strict scientific processes.

First, expert knowledge is presented as legitimate because the proper procedures are used: “I don’t have blind faith in scientists, but I do trust the scientific process, exactly because the process is so reliable. It’s big news when something goes wrong” (TheoM), or “put three scientists together and you have four theories. Their most important activity is constantly gaining new knowledge, and through discussion, arriving at better collective understandings” (Johan_van_Amersfoort). These commenters further suggest that “the beauty of science is that truth will always eventually surface” (W29), exactly because its “repeatable and independent tests and logic” (Bart68). Another commenter adds that researching alternative hypotheses, such as conspiracy theories, is important, “but it should be done in a scientific, verifiable way and not according to the ‘but I just feel that way’ method” (JohanT). These commenters juxtapose expert knowledge with experiential knowledge saying that “feelings are the culprit for many” (JohanT) and that “you can ‘feel’ forever, but if I don’t ‘feel’ exactly the same I won’t learn anything. Without facts you can’t do much” (VanDijk). In theory, they hold, quality knowledge could potentially be constructed by everyone, but “what conspiracy theorists do is ridicule verifiable methods, their motto is ‘if you can’t convince them, confuse them’” (JohanT).

In line with this argument, conspiracy theories are said to lack evidence. Commenters argue that “they are labelled conspiracy theories when their claims can’t be substantiated, but in the end, only facts, and nothing but the facts count” (Stuiter). Unlike “scientists [who] theorize reality on the basis of plausible evidence. If another theory is more plausible they would support that. Progressive insights!” (Klopger). Commenters argue that the difference between scientists and heretics, critical thinkers or whistle-blowers—often alluded to by conspiracy theorists to augment their own epistemic authority—is that “Galileo and Darwin could scientifically substantiate their theories, conspiracy theorists cannot” (Kaya_Dee), and “Snowden and Assange are, of course, the opposite of conspiracy thinkers. They bring hard evidence to the surface. That is precisely what conspiracy thinkers lack” (Rene_Fijnhart).

Others argue that we should allow for alternative ways of knowing beyond rigorous scientific processes, most notably through intuition and experience. As Thomas_Van_der_Langen laments,

Why is “proof” so important to take something seriously? You can also feel something, right? Science is just a perspective, an important, but limited perspective. There is so much that we cannot measure/understand/grasp. Does that necessarily make it “not true”? If truth is only that which the current state of science can prove, then . . . Wow.

They argue that “not everything can be explained with facts” (Gerard_van_der_Molen_efdacc), and highlight

the experience aspect. How can someone live a good life based on facts *alone*? That’s a technicians’ point of view. The experience aspect is the compass on which people sail, and that’s exactly what science hardly sees. (oneshe)

These commenters appreciate other forms of knowledge as well, “I have done a lot of research into different dreams and fantasies which were a revelation to me. I learnt a lot of new things that I couldn’t have learned at school or in the news. And yes, supported by rigorous research” (Patricia_C). Experts may not wish to consider such alternative epistemologies, but that is a missed chance, these commenters hold, because “if they had followed their intuition more often, they would probably look at it differently. Having experiences makes you know something exists” (hs5). Such commenters distinguish between those with “book knowledge [vis-à-vis] practical knowledge to deduce problems and solve them” (Kees_Visser_ccdcbc), arguing that the latter group’s “common sense” (robielienios), “horse sense and survival instinct” (Wil_P_cbdccd), “life-experience and ‘street-wise knowledge’” (Skunk83) are important epistemologies too. In contrast to expert knowledge alone, these people argue that we have many more sources of valuable knowledge, and why not include those?

### Consequences: What is at stake when we let conspiracy theorists in?

The consequences of letting people with diverging views and opinions enter public debate figured prominently. Some people argue that this gives a stage to dangerous fanatics and uninformed people, legitimizes their views, and will lead to undesired tensions between citizens, flawed policymaking, and greater societal harm. On the contrary, keeping an open public debate with as little restrictions as possible is regarded essential in democratic societies. Stigmatization and societal exclusion may only lead to alienation and radicalization.

Commenters advance various experienced interpersonal conflicts with acquaintances who have “conspiratorial” ideas. One described how he has “a conspiracy thinker in the family. She is not stupid but has been through some things. I choose not to bring up conspiracies around her anymore, because it only leads to quarrels” (HansvaHH). Another commenter brings up that “there are people in my network who happen to be in the alternative/spiritual corner. Unfortunately, they are impossible to reason. You are only left to marvel at their positions” (asd_1978). Moreover, such commenters argue that including people with conspiratorial ideas will only motivate them to strengthen their views:

if you take people with completely disturbed and self-invented delusions by the hand and listen to them seriously, this will actually stimulate them to stop looking at the real facts and only see their own ideas as facts. (Kaaselot)

Including conspiracy theorists in public debate may not just legitimize their viewpoints, but may lead to dangerous behaviors as well, such as “inciting people with their invented ideas to violence, or destroying others’ possessions (such as 4&5G-masts). You should not reward them by giving them a stage” (Frank_Sunny). These commenters especially highlight potentially harmful ideas, “if someone says corona is just the flu, climate change does not exist and the Holocaust never happened, you are soon done talking” (Sjors_Koevoets). How can we still speak to each other, these commenters, ask, if we cannot agree on our basic shared reality?

The role of other ways of knowing adds to those fears of losing common ground, some commenters speak of “a schism between people who can’t do anything with feelings, and people who can’t do anything with facts and proof” (Thomas_van_der_Langen). They lament the loss of a “shared reality”:

the difficulty is that there is no common ground. They constantly move the goalposts. Every counter-argument is a “cover-up.” In the meantime, every meme, YouTube video, random blog, is listed as a reliable source. It is up to the other to fight that tangle of misinformation, and because there is no common ground, it is impossible. (W29)

Public discussions with conspiracy theorists are utterly difficult, because “they are unwilling to listen and discuss. Everything is already set in stone. It is just like with orthodox believers: there is no discussion possible, because everything in their eyes is black or white” (Elena_7008). Aside from such communicative problems, commenters also warn that effective governance may be at stake. Including conspiracy thinkers would “sow unfounded doubt and harm and frustrate political decision-making” (PdC). The consensus among these commenters is thus that including citizens with conspiratorial ideas causes both interpersonal and societal problems.

Other commenters defend the notion of an open public debate based on inclusive democratic arguments. The confrontation of a variety of views contributes to critical evaluations of knowledge, they argue, saying that “skepticism advances the world and science” (Rob_van_de_Vliet) and that “a plurality of ideas is critical in advancing insights into how the world works” (TheoM). Ynze_Bakker testifies to this perspective by saying how she is “open to all kinds of opinions, so I can form my own opinion from reviewing all those. I also look at so-called ‘conspiracy theories’ and they often contain better-founded opinions than what I see in the ‘mainstream media.’” According to such commenters, we move forward through an open confrontation of ideas, and all parties, “conspiracy thinkers” or otherwise, can learn from “the other”:

If you don’t take someone seriously and call them a conspiracy theorist without going into discussion, neither of you will get anywhere. He will think you’re wrong and will continue doing his thing, and so will you. While it can be a good learning moment for both. About the point of disagreement and about what led people to these beliefs. (john_west)

This perspective reframes the notion of conspiracy theorists as “trouble” into something more productive, “genius and insanity are, after all, close together” (Willum).

Second, having inclusive public debates that are open to marginalized ideas are seen as a way to prevent alienation and radicalization. Commenters argued, for example, that “if people’s questions are not taken seriously, this results in dogmatic conspiracy thinking, and critical people become messiahs with their own following” (duke_bird). As another commenter explains, “I know from my own experience that ‘not being heard’ can be extremely frustrating. This is probably grossly underestimated. Ignoring someone for a long time can literally kill such a person!” (Pardonlul). These comments contrast sharply with those who fear that inclusive public debates are harmful to society. Instead, they hold, having an arena to openly discuss various ideas would actually prevent such outcomes. “People behave like you treat them” (Eli_Ri). In addition to including alternative positions instead of pathologizing them, this group of commenters advances transparency as an essential ethic in government-citizens relations. “It is difficult to trust the authorities when they are not transparent, trust entails transparency and integrity, and that’s under severe pressure now” (Cooknbacker). To prevent conspiracy theories from thriving, they argue, “governments should be open about the science behind the measures taken. If you keep matters vague and hidden, this is what you get, that’s how critical thinkers become conspiracy thinkers” (Saksaf). Transparency and the inclusion of alternative voices are thus important to keep public debates healthy.

## 5. Conclusion: From post-truth to poly-truth

Post-truth is presented as one of the most defining societal problems of our time. Various (elite) commenters problematize the presence of untruthful information, such as conspiracy theories, in the public sphere (Kakutani, 2018; Lewandowsky et al., 2017; McIntyre, 2018). They lament the (apparent) distrust toward facts, experts, and democratic institutions and wish to remedy these “infodemics” through fact-checking and the removal of dubious contents (Lewandowsky et al., 2020; Zarocostas, 2020). In such analyses, rather clear-cut distinctions are made between the esteemed objective realm of facts, science, and reason and the “dangerous” subjective realm of emotions, ideology, and irrationality. As such, they reinstate the modernist legitimation narrative of science (Harambam, 2021) and profess the classical “politics of demarcation” (Marres, 2018).

But how do everyday people speak about matters of truth and reliable knowledge in the public sphere today? Based on our analysis of a vibrant (Dutch) online discussion on conspiracy theories, we conclude that public opinions show more complexity and nuance than elite conceptions of post-truth allow for. We show that if we listen to a broader public, post-truth is not so much about having moved *beyond* truth, as the dominant definition implies, but instead indicates fierce public battles *about* truth. What is at stake is the rationality of distrusting official knowledge (section “Habitus of distrust: How to appreciate established truths?”), who gets to decide what is true (section “Actors: Who should have a say in public debate?”), which ways of knowing count (section “Knowledge: Which ways of knowing should we allow?”), and what consequences this has (section “Consequences: What is at stake when we let conspiracy theorists in?”). More than a mere “information disorder” (Wardle and Derakhshan, 2017), popular discussions about conspiracy theories lay bare these multiple sociological dimensions of post-truth.

This recognition leads us to interpret the main disagreement in our findings to center around *poly-truth*: the appreciation of multiple actors and forms of knowing. Here, we find clear tensions between technocratic and democratic ideals. On one hand, people argue in favor of a society which trusts authorities, experts, and science. They should have a say in public debates, because scientific experts are well-informed and produce factual knowledge based on strict methodological procedures. Conspiracy theories have no place here because they go against the scientific method and because their proponents are not suitable for reasonable debate. On the other hand, we find democratic arguments favoring a society in which we should not just follow technocratic experts (who can be biased or have exterior motives), but also other people and perspectives, including conspiracy theories/-ists, to account for more epistemic diversity and to have a more inclusive society. These arguments mimic what Mede and Schäfer (2020) describe as “science-related populism”: such commenters indeed critique the “truth-speaking sovereignty” of science in favor of multiple actors and multiple ways of knowing, but they do not downplay the importance of scientific research and experts on the whole, nor does it necessarily come from a populist distrust of elites.

Our analysis highlights an often overlooked tension in contemporary democratic societies: while we value top-quality experts and their knowledge, this conflicts with widespread democratic ideals of inclusion and participation. The one-million-dollar question for the twenty-first century is how to reconcile this issue: how can we include a diverse group of people and ideas, while preserving the merits of expert knowledge? Sophia Rosenfeld (2018) shows the political and epistemological foundations of this tension in her history of post-truth, while sociologist Cathrine Holst (2014) led a research project on knowledge and expertise in modern democracies [EPISTO], which produced an insightful collection of essays on the topic. Philosopher M. Dentith (2018), political-theorist Alfred Moore (2017) and sociologist Jaron Harambam (2021) independently draw on deliberative political theory and social studies of science to develop ways of institutionalizing popular interrogations of expert knowledge in a societally productive way. Similar “citizen assemblies” showed their success in recent experiments across the world (Farrell et al., 2019; Smith, 2009). We see more future in these efforts than foreclosing public discussions about truthful knowledge by empty appeals to the authority of facts and science, especially as such citizen platforms may prevent radicalization, polarization, and even more distrust of our epistemic authorities. Whereas pluralism, or its contemporary buzzwords of diversity and inclusion, is desired in so many societal domains today, we wonder why this is not the case with knowledge. Our analysis showed public opinions on both the merits and dangers of poly-truth. Perhaps that above-mentioned organized forms of including various people, epistemologies, and interests in public debates would tame (elitist) post-truth fears of a misinformed and irrational mob stealing Truth from its pedestal?

## Notes

1. Our data set is openly available in *Harvard Dataverse* at https://doi.org/10.7910/DVN/BW2DLW.
